# *In vitro* and *in vivo* Effects of a Single Dose of Bupivacaine 5% on Donkey Chondrocytes

**DOI:** 10.3389/fvets.2021.661426

**Published:** 2021-11-23

**Authors:** Kamal Hussein, Abdelbaset Eweda Abdelbaset, Ahmed Abdelrahiem Sadek, Ahmed Noreldin

**Affiliations:** ^1^Department of Animal Surgery, Anaesthesia, and Radiology, Faculty of Veterinary Medicine, Assiut University, Asyut, Egypt; ^2^Clinical Laboratory Diagnosis, Department of Animal Medicine, Faculty of Veterinary Medicine, Assiut University, Asyut, Egypt; ^3^Department of Histology and Cytology, Faculty of Veterinary Medicine, Damanhour University, Damanhour, Egypt

**Keywords:** bupivacaine, local anesthetic, intra-articular, synovial fluid, cartilage damage, chondrolysis, donkey

## Abstract

Single intra-articular (IA) injection of long-acting local anesthetics such as bupivacaine is commonly used clinically for postoperative analgesia, in particular, after arthroscopic surgery. Despite their widespread use, the side effects of IA bupivacaine on joint cartilage as well as hepatotoxic and nephrotoxic effects remain to be elucidated. The aim of this study is to assess the *in vitro* effect of bupivacaine 5% on donkey chondrocytes at different time points, in addition to the *in vivo* effects of a single IA bupivacaine injection on the middle carpal joint in a group of 10 clinically healthy adult male donkeys. In phase I, the effect of *in vitro* treatment with bupivacaine 5% or saline 0.9% on freshly isolated donkey chondrocytes for 30, 60 min, 24, 48, and 96 h was investigated using MTT and LIVE/DEAD assay. In phase II, *in vivo* effects of single injection of bupivacaine on the middle carpal joint of the donkey were evaluated compared with saline 0.9%. Biochemical analysis of collected serum and synovia was performed. Additionally, articular cartilage damage was evaluated using radiography, computed tomography (CT), catabolic marker expression *via* quantitative polymerase chain reaction (qPCR), and histopathological examination 96 h after injection. Our results showed that after a 30-min exposure to bupivacaine 5%, the viability of donkey chondrocytes was 97.3 ± 4.4% and was not significantly affected at the indicated time points (*n* = 8, *p* < 0.05). No significant changes in biochemical analytes of serum and synovial fluid following IA bupivacaine injection were observed, compared with saline injection (*n* = 5 for each group, *p* < 0.05). Furthermore, *in vivo* IA injection of bupivacaine revealed no significant differences in radiography, CT scan, gene expression of cartilage catabolic biomarkers, and histopathological examination. These results provide an evidence for the safety of bupivacaine on the donkey cartilage.

## Introduction

Arthroscopic knee surgery is a commonly performed orthopedic surgery (1, 2). Postoperative pain and delay of recovery are common consequences of such surgery in humans and animals (3, 4). Single intra-articular (IA) administration of long-acting local anesthetics is a widely utilized approach for pre- and post-operative analgesia after arthroscopic surgeries due to simplicity and low economic costs (5). Despite their extensive use, concerns of chondrotoxicity have been recently raised especially for bupivacaine, levobupivacaine, ropivacaine, mepivacaine, and lidocaine (6–9).

*In vivo* and *in vitro* effects of IA bupivacaine on chondrocytes have been varied with some reports showing chondrotoxicity while others showing no adverse effects (10–18). For instance, Park et al. have suggested that IA administration of bupivacaine may induce a marked chondrotoxicity based on *in vitro* viability results after exposure of equine chondrocytes to bupivacaine 0.5% (18). On the contrary, *in vivo* IA injection of bupivacaine was reported to have anabolic effects on equine cartilages, as evidenced by a significant increase in markers of cartilage matrix synthesis and a decrease in markers of collagen degradation (17). In the latter study, the authors hypothesized that the lack of chondrotoxicity observed *in vivo*, compared with that noticed in cell culture (18), might be attributed to dynamic changes of bupivacaine concentrations in joints (17).

Different studies have identified additional biomarkers of cartilage matrix degradation including matrix metalloproteinases (MMPs), metallopeptidase with thrombospondin type 1 motifs (ADAMTS), and cartilage oligomeric matrix protein (COMP). Such biomarkers could aid in the detection of early toxic effects of IA injection of therapeutics on the cartilage *in vivo* (19, 20). To our knowledge, the effect of IA bupivacaine injection on these catabolic markers of the articular cartilage has not been elucidated yet. Hence, the present study aimed to investigate the possible changes in the gene expression of those cartilage damage biomarkers following IA bupivacaine injection. Moreover, the study also aimed to analyze the serum amyloid A (SAA) level (as an inflammatory marker), hepatic enzyme activity, blood urea nitrogen (BUN), and creatinine levels. Finally, radiographic examination, computed tomography (CT) scanning, and histopathology were performed to explore the effect of bupivacaine on the cartilage of the middle carpal joint.

## Materials and Methods

The study protocol was designed according to the Guidelines for Animal Experimentation and with the approval of the Committee of Research Facilities, Faculty of Veterinary Medicine, Assiut University, Egypt, and was conducted in accordance with the Animal Research: Reporting of *In Vivo* Experiments (ARRIVE) guidelines. The current study was divided into two phases. The first phase of the study (phase I), *in vitro* experiments, was performed to check the effect of bupivacaine on the viability of donkey chondrocytes. The second phase of the study (phase II), *in vivo* experiments, was done by injection of bupivacaine or saline in the middle carpal joint of 10 donkeys.

### Donkey Chondrocyte Isolation

Primary culture of chondrocytes obtained from articular cartilage was aseptically harvested from the metacarpophalangeal joints of a male donkey (3 years old with a body condition score of 5), euthanized for reasons unrelated to this study. Briefly, nine pieces of 0.5 cm^2^ with 2 mm in thickness from the cartilage surface covering the distal metacarpus, proximal phalanx, and proximal medial and lateral sesamoid bones were rinsed in Dulbecco's phosphate-buffered saline without Ca^+2^ and Mg^+2^ (D-PBS). After enzymatic digestion in 0.25% collagenase type I (Sigma-Aldrich, MO, USA) in high-glucose Dulbecco's modified Eagle's medium (DMEM; Invitrogen, CA, USA) containing 10% heat-inactivated fetal bovine serum (FBS; Hyclone, Logan, UT, USA), the cell suspension was filtered to remove undigested cartilage fragments. The filtered fluid was centrifuged at 100 × *g* for 5 min, then the supernatant was discarded. The cell pellet was resuspended in high-glucose DMEM containing 10% FBS and 1% penicillin/streptomycin. The cell suspension was cultured in a 5% CO_2_ and humidified atmosphere at 37°C until formation of monolayers suitable for propagation. The chondrocytes of the first passage were used in the various assays.

### Viability Assay

A suspension of 30 × 10^3^ donkey chondrocyte (counted by a hemocytometer) was cultured in 48-well plates (SPL Life Sciences Co., South Korea) and was divided into four treatment groups. Each group was treated with one of the following experimental agents to 200 μl of culture media: (1) 200 μl of saline solution 0.9% (Elnasr Pharmaceuticals Chemicals Co., Cairo, Egypt), (2) 200 μl of bupivacaine 5% (Sunnypivacaine, Sunny medical Co., Cairo, Egypt), (3) untreated cells as a negative control group, and (4) dimethyl sulfoxide 20% (DMSO; Merck, Darmstadt, Germany) as a positive toxic group, for 30, 60 min, 24, 48, and 96 h. At different time points, 3-(4,5-dimethylthiazol-2-yl)-2,5-diphenyltetrazolium bromide (MTT) assay was performed (21). Briefly, 50 μl of MTT solution (5 mg ml^−1^; Sigma-Aldrich) was added to each well and incubated at 37°C for 4 h. After discarding the medium containing MTT, 250 μl of DMSO was added to all wells to dissolve the formazan into a purple solution. After a 10-min incubation, 100-μl aliquots from the wells were pipetted into another 96-well plate (SPL Life Sciences Co.). The newly developed color was quantified by recording the absorbance at a wavelength of 570 nm with a spectrophotometer. The cell activity was represented as the percentage of activity expressed by cells compared with the negative control.

To qualitatively assess the cell viability after 96 h, staining with a LIVE/DEAD assay kit (Calcein-AM/ethidium bromide homodimer; Invitrogen) was performed, and imaging using a fluorescence microscope (Olympus, Tokyo, Japan) was performed.

### *In vivo* Study in Donkeys

A group of 10 clinically healthy adult male donkeys (1.5–2 years old) weighing 265 ± 27 kg (mean ± standard deviation) was included in the experiment. The animals were housed at the Veterinary Teaching Hospital, Assiut University, and were fed twice daily. Before the study, the animals were examined clinically and radiographically to exclude any abnormal condition of the right carpal joint. The right carpal joint of each donkey was used in this experiment to evaluate the *in vivo* changes following IA administration of bupivacaine HCl 5%. The donkeys were randomly and equally (online randomization website http://www.randomization.com) assigned to two groups (five animals for each group); the first group received bupivacaine HCl 5%, while the second group received saline 0.9% as a negative control group. Group assignments were concealed in sealed envelopes.

### Intra-articular Injection

The intra-articular (IA) injection was performed as previously described (8, 17) after sedation of animals with xylazine HCl 2% (Xyla-Ject, ADWIA Co., Cairo, Egypt) in a dose of 2 mg kg^−1^ body weight. Aseptic preparation of the injection site was performed by scrubbing the skin area over the right carpus using povidone iodine, then sprayed by alcohol 75%. The carpal joint was flexed, and a sterile 20-gauge needle was inserted into the middle carpal joint, then 5 ml of bupivacaine HCl 5% or saline solution 0.9% was injected. Animals were evaluated daily during the study period for any signs of hyperthermia, changes in the heart and respiratory rates, joint effusion, abnormal gait, and pain in the injected limbs.

### Blood Collection and Analysis

Whole blood samples of the donkeys were collected from the jugular vein, in 5-ml plain vacutainer tubes (Biomedica Alex Co., Alexandria, Egypt) before IA injection, and 24, 48, and 96 h after injection. The samples were centrifuged for 15 min at 1,500 × *g* speed, and sera were then harvested and preserved at −20°C until used. Serum levels of alanine aminotransferase (ALT), aspartate aminotransferase (AST), alkaline phosphatase (ALP), BUN, and creatinine levels were measured *via* commercial test kits (Spinreact, Girona, Spain), using a UV spectrophotometer (Optizen 3220 UV, Mecasys Co. Ltd, South Korea).

### Synovial Fluid Analysis

Synovial fluid (SF, 1 ml) was obtained from the right middle carpal joint prior to drug injection, and 24-, 48-, and 96-h postdrug injection. The site for arthrocentesis was aseptically prepared. The carpal joint was flexed followed by aspiration of SF with a sterile needle on heparinized vacutainer tubes (Biomedica Alex Co.). SF was mixed thoroughly to avoid any coagulation and used for biochemical analysis. SAA was analyzed using donkey SAA ELISA Kit (MyBiosource, San Diego, CA, USA). Also, the biochemical constituents of SF including AST, gamma-glutamyl transferase (GGT), lactate dehydrogenase (LDH), and total proteins were measured *via* commercial test kits (Spinreact), using a UV spectrophotometer.

### Radiographic Evaluation

Lateromedial and dorsopalmar radiographs were taken for six donkeys (*n* = 3 for each group) to evaluate the articular cartilage damage at 96 h post-injection using a fixed X-ray apparatus (50 kV and 15 mA s^−1^) (Philips Super 80 CP, Germany). Animals were restrained in lateral recumbency under the effect of sedation with xylazine HCl 2%.

### Computed Tomography Scanning

Euthanasia of six donkeys (*n* = 3 for each group, randomly selected using online randomization website http://www.randomization.com) by intravenous injection of xylazine HCl 2% (1 mg kg^−1^) followed by sodium thiopental (40 mg kg^−1^, Sandoz GmbH, Kundl, Austria) was performed according to the AVMA instructions (22) 96 h after IA injection. Euthanasia was approved by the Committee of Research Facilities, Faculty of Veterinary Medicine, Assiut University, Egypt. The right forelimbs were separated at the level of the shoulder joint. Computed tomography of the carpal joint using a Philips 128 slice scanner apparatus was performed (*n* = 3 for each group). Sagittal, dorsal, and transverse images were obtained, and the distance between the slices taken was 0.5 cm.

### Quantitative Polymerase Chain Reaction

We investigated the possible effects of bupivacaine on the gene expression of different catabolic markers including MMP-3, MMP-13, ADAMTS-5, and COMP. After euthanasia, total RNA was isolated from chondrocytes of the harvested joints (*n* = 3 for each group) and transcribed into cDNA using the NucleoSpin RNA Mini kit (Macherey-Nagel GmbH & Co., Germany) and TOPscrip RT DryMIX (Enzynomics, South Korea), respectively. Quantitative real-time polymerase chain reaction (PCR) was performed using TOPreal qPCR 2 × PreMIX (Enzynomics) on a StepOnePlus real-time PCR system (Thermo Fisher Scientific) following the recommendations of the manufacturer. The relative gene expression was calculated by the comparative Ct (2^−Δ*ΔCt*^) method with glyceraldehyde 3-phosphate dehydrogenase (GAPDH) as an internal control. The primer sequences used for qPCR are listed in Table 1.

**Table 1:** Sequences for quantitative polymerase chain reaction (qPCR) primers.

**Primer**	**Sequences**	**Accession number**
Matrix metallopeptidase 3 (MMP-3)	F5′-GCAAGGGACGAGGATAGCAA-3′ R5′-TGCATCACCTCCACAGTGTC-3′	XM_014860021.1
Matrix metallopeptidase 13 (MMP-13)	F5'-AGAAGACTGCAGCGAACTCC-3′ R5′-TGCCAGTCACTTCTAAGCCG-3	XM_014860022.1
ADAM metallopeptidase with thrombospondin type 1 motif 5 (ADAMTS5)	F5′-CGAGCGGGAAAGAGTTGTG-3′ R5′-ACTCCATCCACCAGCAAACG-3′	XM_014866273.1
Cartilage oligomeric matrix protein (COMP)	F5′-GAACTGCAAGAGACCAACGC-3′ R5′-GTCACACTCCATCACCGTGT-3′	XM_014865279.1
Glyceraldehyde-3-phosphate dehydrogenase (GAPDH)	F5′- CTTGTCTCCCTCAGATTTGGC-3′ R5′-AAGGGGTCATTGATGGCGAC-3′	XM_014866500.1

### Histological Examination

Full thickness articular cartilage samples were surgically obtained from the right middle carpal joints with a diameter of 5 mm. Sampling sites were chosen to represent varying areas of thick and thin cartilage regions. Then the samples were flushed with PBS and fixed in neutral-buffered formalin for 48 h. The fixed specimens were decalcified by immersing in EDTA solution for 3 weeks and then processed by the conventional paraffin embedding technique. Sections of 4-μm thickness were stained by hematoxylin and eosin (H&E) according to the method described by Bancroft and Layton (23).

### Statistical Analysis

Results are presented as means ± standard deviations. Statistical analysis of the data by two-way ANOVA followed by Tukey's HSD *post-hoc* test was performed using SPSS software (Version 21; IBM Corp., Armonk, NY, USA). The results of the qPCR were analyzed by Student's unpaired *t*-test. Values of *p* < 0.05 were considered significant. A sample size of 10 donkeys was used as this was the maximum number of animals able to be accommodated with the available resources.

## Results

### *In vitro* Effect of Bupivacaine on the Donkey Chondrocytes

**Figure 1:**
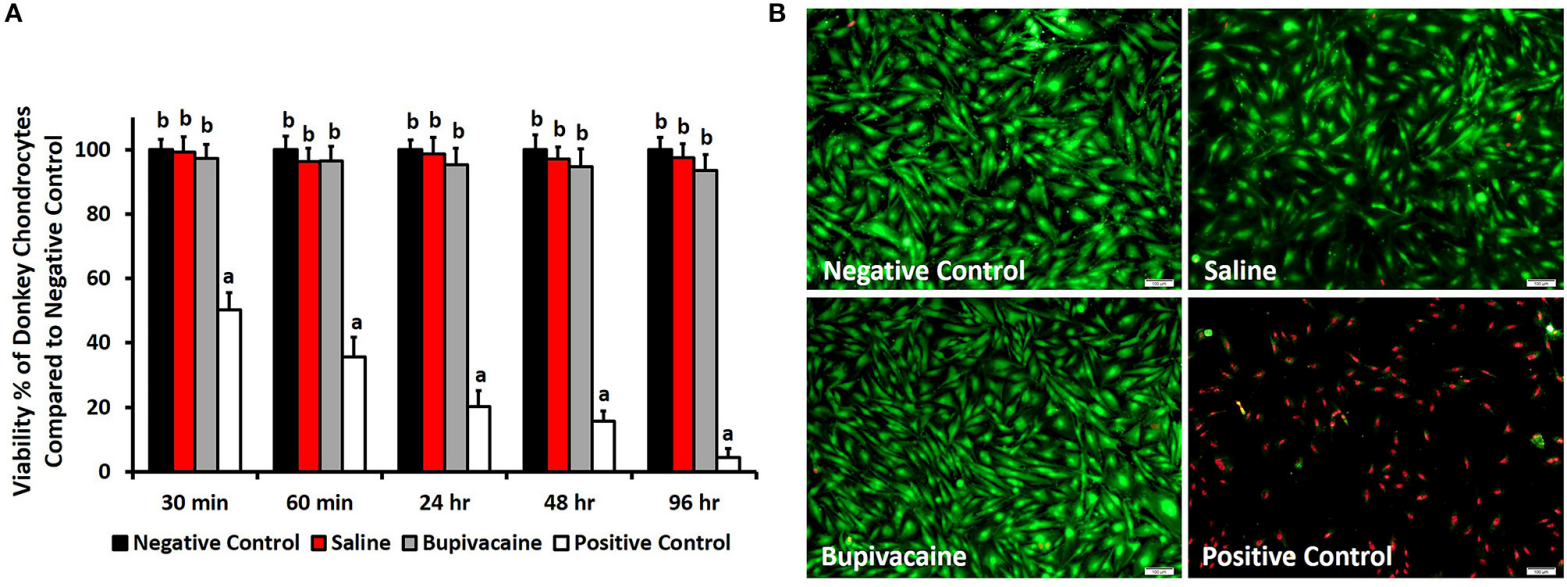
*In vitro* viability testing. **(A)** 3-(4,5-Dimethylthiazol-2-yl)-2,5-diphenyltetrazolium bromide (MTT) assay for evaluating the viability of donkey chondrocytes after exposure to saline 0.9% or bupivacaine 5% for 30, 60 min, 24, 48, and 96 h. Error bars represent means ± standard deviation (*n* = 8). **(B)** Cell viability using LIVE/DEAD assay for donkey chondrocytes after exposure to normal saline 0.9% or bupivacaine 5% for 96 h. Live cells were stained green, while dead cells were stained red (magnification = ×10).

The MTT assay results (Figure 1A) revealed that the percentage of live donkey chondrocytes in the group subjected to a 30-min exposure to bupivacaine 5% was 97.3 ± 4.4% and was not significantly affected at the different time points (*p* < 0.05). Saline 0.9% group did not display any cytotoxic effect on donkey chondrocytes. Only a few cells of donkey chondrocytes were positive to EthD-1 staining after exposure to saline or bupivacaine, as shown in Figure 1B, which shows the cell viability after 4 days of culture, indicating that viability was maintained over time upon culture of cells. The results showed that DMSO had a significant toxicity on the donkey chondrocytes (Figure 1B).

### Physical Examination Findings

Clinical examination of donkeys showed no fever or changes in the heart and respiratory rates. No abnormal gait was observed in donkeys during the study. Moreover, no joint effusion or swelling after IA injections of bupivacaine 5% or saline 0.9% were recorded.

### Biochemical Analysis of Serum

As shown in Table 2, biochemical analysis of serum ALT, AST, ALP, BUN, and creatinine levels revealed a non-significant difference in animals receiving an IA injection of bupivacaine 5% compared with animals injected with saline 0.9% (*p* < 0.05).

**Table 2:** Biochemical analysis of serum.

**Parameter**	**Group**	**Day 0 (before IA injection)**	**24-h post-IA injection**	**48-h post-IA injection**	**96-h post-IA injection**
ALT (U/L)	Saline	13.5 ± 4.95	16 ± 5.65	15.07 ± 6.97	16.75 ± 3.88
	Bupivacaine	12.33 ± 2.88	18.66 ± 7.50	17.07 ± 3.89	11.13 ± 4.12
AST (U/L)	Saline	228.15 ± 2.41	217.02 ± 39.56	206 ± 11.31	227.5 ± 21.92
	Bupivacaine	217.5 ± 12.25	226.33 ± 3.055	219.33 ± 8.08	225.66 ± 11.01
Alkaline phosphatase (U/L)	Saline	105.5 ± 13.43	101 ± 21.21	98 ± 12.82	105 ± 11.31
	Bupivacaine	129 ± 8.07	114.33 ± 10.62	108 ± 19.07	103.66 ± 22.18
Urea (mg/dl)	Saline	20 ± 9.89	19 ± 2.82	21 ± 8.56	17.8 ± 4.69
	Bupivacaine	21 ± 7.21	17.63 ± 9.23	21.33 ± 4.93	15.66 ± 4.16
Creatinine (mg/dl)	Saline	1.14 ± 0.07	1.1 ± 0.28	1.42 ± 0.39	1 ± 0.28
	Bupivacaine	1.15 ± 0.15	1.19 ± 0.09	1.1 ± 0.2	1.36 ± 0.38

*Serum samples were analyzed for ALT, AST, alkaline phosphatase, urea, and creatinine after intra-articular (IA) injection of bupivacaine 5% or normal saline 0.9% (n = 5 for each group).*

*ALT, alanine aminotransferase; AST, aspartate aminotransferase*.

### Synovial Analysis

All parameters used for evaluation of synovia including SAA, AST, GGT, LDH, and total proteins, showed non-significant differences in donkeys injected by bupivacaine 5% when compared with those injected with saline 0.9% (Table 3) (*p* < 0.05).

**Table 3:** Synovial fluid (SF) analysis.

**Parameter**	**Group**	**Day 0 (before IA injection)**	**24-h post-IA injection**	**48-h post-IA injection**	**96-h post-IA injection**
SAA (μg/mL)	Saline	2.92 ± 2.39	5.14 ± 1.78	5.63 ± 2.21	3.36 ± 2.58
	Bupivacaine	2.74 ± 2.51	4.92 ± 2.1	5.03 ± 3.26	2.98 ± 2.08
AST (U/L)	Saline	12.72 ± 7.97	21.53 ± 17.15	12.76 ± 9.35	15.66 ± 4.16
	Bupivacaine	15.5 ± 12.28	18.14 ± 12.34	10.99 ± 9.86	14.03 ± 12.26
GGT (U/L)	Saline	68.75 ± 36.85	83.46 ± 33.72	65.34 ± 34.70	70.41 ± 31.82
	Bupivacaine	65.4 ± 31.44	74.8 ± 34.14	61.2 ± 38.89	60.57 ± 37.69
LDH (U/L)	Saline	109.66 ± 5.78	135.25 ± 31.19	103.76 ± 15.61	102 ± 16.09
	Bupivacaine	120.8 ± 22.34	166.66 ± 60.16	130.4 ± 71.65	98.01 ± 67.2
Total protein (g/dL)	Saline	16.43 ± 13.35	7.16 ± 5.18	9.36 ± 2.08	6.13 ± 2.8
	Bupivacaine	6.23 ± 5.74	11.48 ± 7.23	13.88 ± 11.85	8.89 ± 7.92

*SF samples were tested for AST, GGT, LDH, total protein, and SAA levels after IA injection of bupivacaine 5% or normal saline 0.9% (n = 5 for each group).*

*SAA, serum amyloid A; AST, aspartate aminotransferase; GGT, gamma-glutamyl transferase; LDH, lactate dehydrogenase*.

### X-Ray and Computed Tomography Scanning

**Figure 2:**
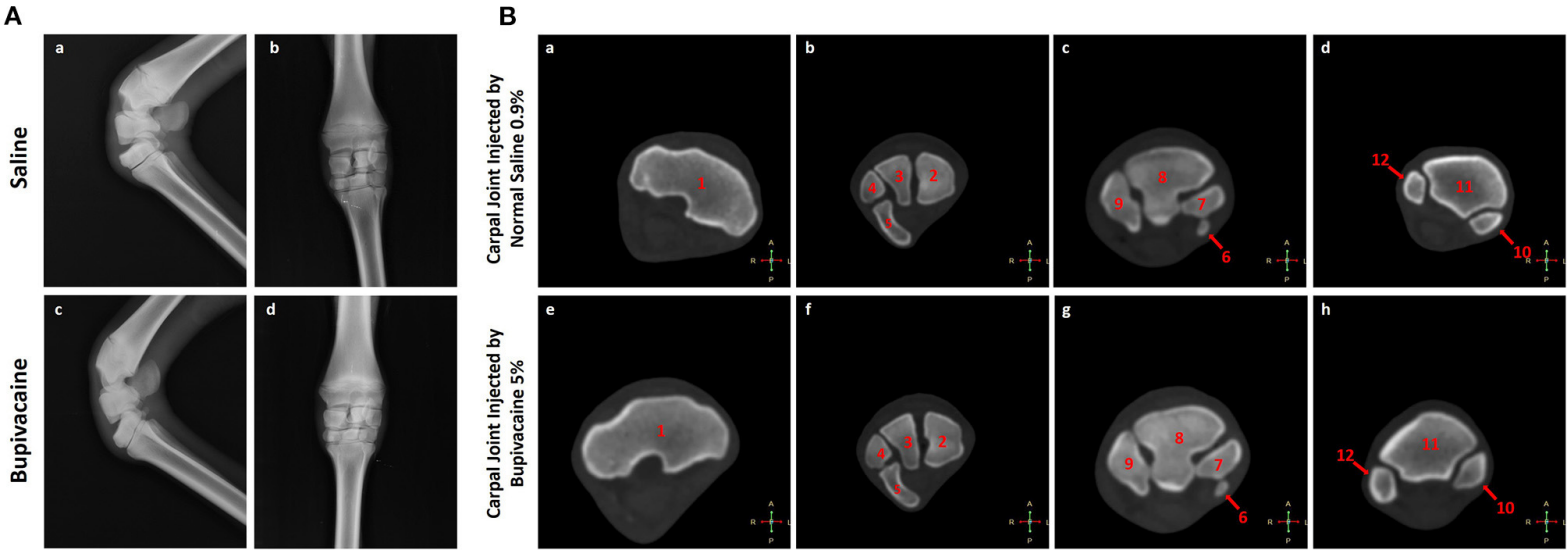
Imaging of the carpal joint. **(A)** Radiography of the carpal joint after 96 h of saline 0.9% or bupivacaine 5% IA administration. (a,c) Latero-medial view, and (b,d) anteroposterior view. **(B)** Transverse computed tomography bone window for the carpal joint after 96 h of saline 0.9% or bupivacaine 5% IA administration. (a,e) Transverse CT scans at the level of the trochlea radii. (b,f) Transverse CT scans at the level of the proximal carpal row. (c,g) Transverse CT scans at the level of the distal carpal row. (d,h) Transverse CT scans at the level of the proximal part of the metacarpus. 1. The distal extremity of the radius. 2. Radial carpal bone. 3. Intermediate carpal bone. 4. Ulnar carpal bone. 5. Accessory carpal bone. 6. First carpal bone. 7. Second carpal bone. 8. Third carpal bone. 9. Fourth carpal bone. 10. Third metacarpal bone. 11. Second metacarpal bone. 12. Fourth metacarpal bone.

None of the animals treated with bupivacaine 5% or saline 0.9% in their carpal joints demonstrated significant radiographic changes (Figure 2A). This was also confirmed by CT scan (Figure 2B) that demonstrated normal cartilage morphology without chondrolysis or loss of the cartilage layer, with a similar joint space. These results were congruent with the clinical examination of the animals that revealed no joint effusion or swelling after IA injections of bupivacaine 5% or saline 0.9%.

### Quantitative Polymerase Chain Reaction

**Figure 3:**
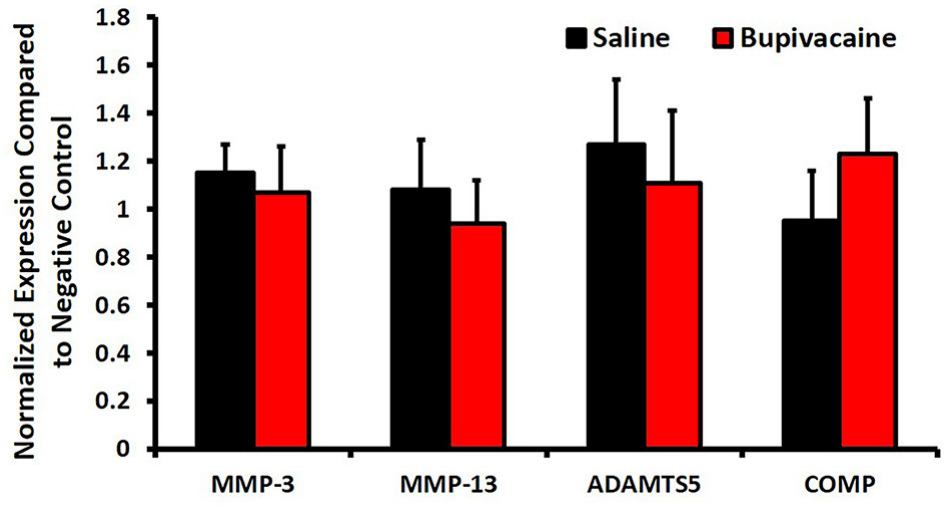
The catabolic activity after IA of bupivacaine injection. The mRNA expression of catabolic factors, matrix metalloproteinases (MMP)-3, MMP-13, ADAM metallopeptidase with thrombospondin type 1 motif 5 (ADAMTS5), and cartilage oligomeric matrix protein (COMP) was measured by quantitative real-time polymerase chain reaction (PCR). Glyceraldehyde-3-phosphate dehydrogenase (GAPDH) was used as an internal control. Bars represent means ± SD, *n* = 3 for each group.

The qPCR results (Figure 3) showed that the gene expressions of MMP-3, MMP-13, ADAMTS-5, and COMP in the saline 0.9% group were 1.15 ± 0.12-fold, 1.08 ± 0.21-fold, 1.27 ± 0.27-fold, and 0.95 ± 0.21-fold, whereas in the bupivacaine 5% group, the gene expressions were 1.07 ± 0.19-fold, 0.94 ± 0.18-fold, 1.11 ± 0.3-fold, and 1.23 ± 0.23-fold, respectively, with no significant differences with the negative control group (normal donkey chondrocytes), confirming the absence of the chondrolysis process and cartilage damage.

### Histological Evaluation

**Figure 4:**
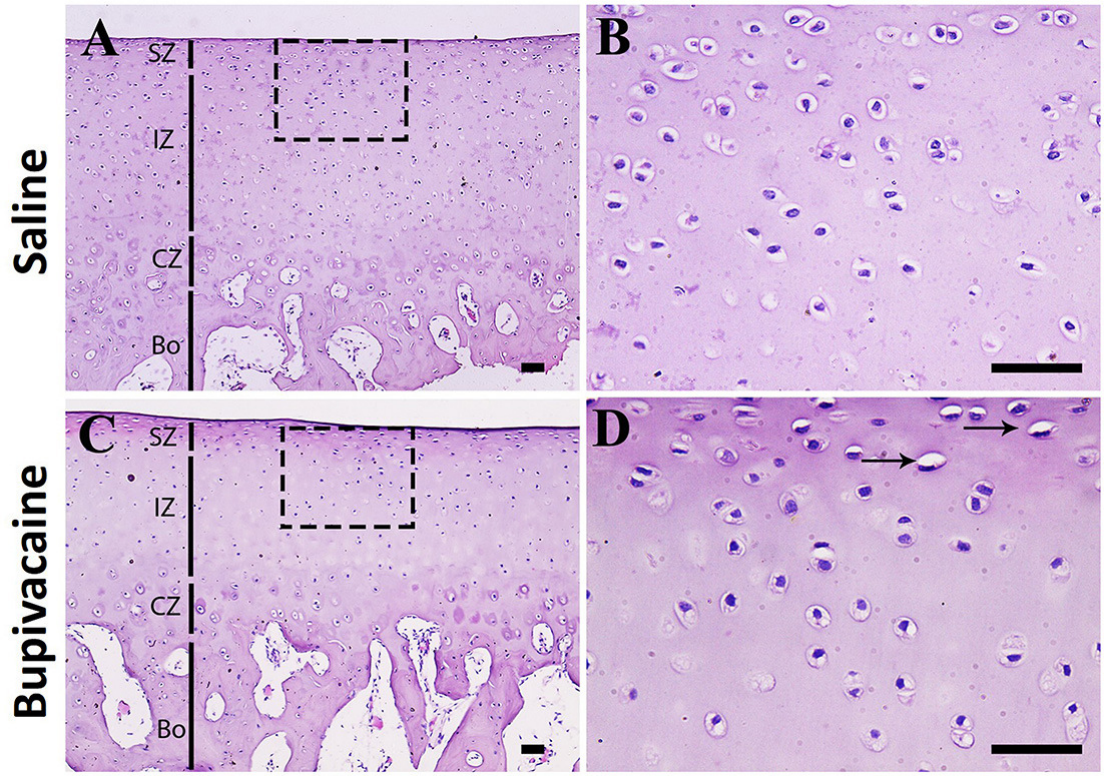
Histopathological examination of donkey cartilage. **(A)** Histological samples of carpal joint injected by normal saline 0.9% showing the normal cartilage architecture. Magnified superficial zone and intermediate zone showing that the chondrocytes are well-arranged **(B)**. **(C,D)** Joints injected by bupivacaine 5% showed similar architecture to control saline group cartilage with few chondrocytes degeneration (arrows). SZ, superficial zone; IZ, intermediate zone; CZ, calcified zone; Bo, bone area. Scale bar = 50 μm, *n* = 3 for each group.

Histopathological examination of donkey cartilage revealed well-arranged chondrocytes in bupivacaine 5% treated joint compared with joints injected by saline 0.9%, confirming the harmless effect of bupivacaine injection in the carpal joint of the donkey (Figure 4).

## Discussion

The purpose of this study was to evaluate the safety of IA injection of bupivacaine in donkeys. To this purpose, we conducted an *in vitro* assay to evaluate the cytotoxic effect of bupivacaine on donkey chondrocytes as phase I. Additionally, we investigated the safety of bupivacaine on donkey cartilage *in vivo* after a single IA administration as phase II. Therefore, we concluded that bupivacaine appeared to be safe on the donkey chondrocytes.

Quero et al. have reported that bupivacaine may have either proliferative effects or concentration-dependent cytotoxicity, upon addition to the culture of human degenerate intervertebral discs. They observed that a dose of 0.05% bupivacaine enhanced the cell proliferation up to 170%, but higher concentrations (≥0.1%) resulted in a severe chondrotoxicity with almost 100% cell death (24). Our findings detected no effect for bupivacaine on the donkey chondrocytes. The mechanisms investigating the chondro-proliferative or chondrotoxic effects of local anesthetics including bupivacaine have not been explained yet. Bogatch et al. have suggested that the cellular effects of the compounds may be attributed to the indirect physical or chemical interactions with the culture medium constituents causing formation of needle-like crystals and resulting in chondrocyte death (25). They detected <5% of bovine chondrocyte death when the anesthetics (lidocaine or bupivacaine) were instilled to chondrocytes alone or in PBS-diluted solution. However, when lidocaine was mixed with culture medium or SF, 99.2 and 96.3% of chondrocyte death occurred, respectively (25). In our study, no significant toxic effect was observed when bupivacaine 5% was mixed with media.

Bupivacaine is extensively metabolized by the hepatic cytochrome P450 system and excreted by the kidneys. Neurological and cardiovascular complications are the main side effects associated with amide local anesthetics; however, bupivacaine-induced liver injury and cholestasis have been reported with epidural or local administration for the treatment of chronic or postoperative pain (26, 27). Lee and Kwak described the occurrence of acute liver injury as a significant complication associated with IA bupivacaine administration after bilateral total knee arthroplasty in an old man with a history of liver transplantation. Hepatic enzyme levels of AST and ALT increased to more than 10 times the upper reference range after the conclusion of surgery and remained enormously high during the postoperative period (28). Lee et al. reported that continuous epidural infusion of bupivacaine 1% resulted in impaired renal functions at day 1 and day 2 after ischemia–reperfusion injury in rats (29). To date, extensive evaluation of hepatotoxicity or renal toxicity in equine due to bupivacaine has not been performed. To explore whether the IA injection of bupivacaine has a systemic effect, we analyzed serum levels of ALT, AST, ALP, BUN, and creatinine. Our findings revealed no significant changes in those analytes, indicating that bupivacaine 5% is non-toxic at this concentration for the liver and kidneys in donkeys.

Serum amyloid A (SAA), a major acute-phase protein, was suggested to be locally synthesized in the inflamed joint of horses (30). It was also reported to be elevated in SF from inflamed synovial structures (31). In addition, serum enzyme activities of ALP, LDH, and creatine kinase (CK) were elevated significantly in acute equine arthritis (32). Total protein concentration, AST, ALP, and LDH were reported to be increased significantly in SF of horses with acute and chronic arthritis and also in response to lipopolysaccharide-induced synovitis in horses (32, 33). In the current study, no significant differences were detected in the SF composition of SAA, AST, GGT, LDH, and total protein levels following single IA bupivacaine injection compared with the control group, confirming the safe effect of bupivacaine on synovia.

Cartilage matrix degradation is associated with the release of MMPs. MMPs, concomitant with ADAMTS, induce degradation of all extracellular matrix constituents including COMP. MMP-3 and MMP-13 were increased in experimental animal models of osteoarthritis (OA) as well as in human OA cartilage (34–36). MMP-13 overexpression in a murine model resulted in a phenotype with articular cartilage destruction (37). Early stages of articular cartilage degeneration were accompanied by upregulation of COMP mRNA expression in a transgenic mouse model of OA (38). Furthermore, knockout of the ADAMTS-5 gene prevented cartilage degradation in a murine knee OA model (39). Recently, Moon et al. demonstrated that the mRNA expressions of MMP-3, MMP-13, and ADAMTS-5 were significantly attenuated in IL-1β-stimulated human OA chondrocytes, using ursodeoxycholic acid (40). Our results revealed no significant changes in the expressions of MMP-3, MMP-13, ADAMTS-5, and COMP between bupivacaine and saline-injected donkeys. These results were in the same line with other investigators who indicated that bupivacaine has no catabolic effect (17). Future investigation of markers of articular cartilage damage or inflammation markers, such as inflammatory cytokines or nuclear factor kappa B (NFkB), cyclooxygenase-2 (COX-2), nitric oxide synthase-2 (NOS-2), interleukin-1β (IL-1β), and tumor necrosis factor-α (TNF-α) should be considered (41, 42).

A histologic assessment of the articular cartilage was performed, and no pathologic effects on the donkey cartilage were detected, contrary to previous *in vivo* investigations that reported articular cartilage chondrolysis in rats (43) and rabbits (44). Other reports have detected severe chondrolysis after continuous IA infusions of bupivacaine and not in case of single injections of local anesthetics (45). Single IA administration of bupivacaine has been used in human and equine joints without clinically apparent chondrotoxic effects for many years. However, this does not confirm that there is no subtle negative effect of single IA injection on diseased articular cartilage as others have shown (12, 16, 18, 43).

There are several limitations in the present study. First, the assessment was performed on normal healthy joints. The same doses of local anesthetics injected into osteoarthritic or inflamed joints could potentially have different effects. Second, the effects of a single dose of each compound were evaluated *in vivo*. The dose that we administered was based on clinically used doses and not on relative potency that remains unknown with respect to IA use of local anesthetics. As dose-dependent toxicity has been previously established with bupivacaine, increased doses may also have enhanced effects (10). Finally, CT arthrography is considered as the best option with high sensitivity for cartilage damage detection in the equine (46); however, we used plain CT for detection of the cartilage damage. Similarly, plain X-ray gave a limited information about cartilage damage in our study. Plain X-ray was reported to remain as a “surrogate marker” for indirect evaluation of cartilage destruction, even with the rapid progress in imaging techniques (47).

In conclusion, the results of the *in vitro* assay and *in vivo* IA injection suggest the safety of a single dose of bupivacaine on the donkey cartilages up to 96 h.

## Data Availability Statement

The original contributions presented in the study are included in the article/supplementary material, further inquiries can be directed to the corresponding author/s.

## Ethics Statement

The study protocol was designed according to the Guidelines for Animal Experimentation and with the approval of the Committee of Research Facilities, Faculty of Veterinary Medicine, Assiut University, Egypt and conducted in accordance with the Animal Research: Reporting of In Vivo Experiments (ARRIVE) guidelines.

## Author Contributions

KH designed and conducted the study, acquired and interpreted the data, performed the statistical analysis, wrote the manuscript, and reviewed the analysis of the data. AA and AS acquired and analyzed the data and wrote the manuscript. AN acquired and interpreted the data and wrote the manuscript. All authors reviewed the manuscript and approved the final version for publication.

## Conflict of Interest

The authors declare that the research was conducted in the absence of any commercial or financial relationships that could be construed as a potential conflict of interest.

## Publisher's Note

All claims expressed in this article are solely those of the authors and do not necessarily represent those of their affiliated organizations, or those of the publisher, the editors and the reviewers. Any product that may be evaluated in this article, or claim that may be made by its manufacturer, is not guaranteed or endorsed by the publisher.

## Abbreviations

MMPs: matrix metalloproteinases
ADAMTS: metallopeptidase with thrombospondin type 1 motifs
COMP: cartilage oligomeric matrix protein
SAA: serum amyloid A
CT: computed tomography
BUN: blood urea nitrogen
DMEM: Dulbecco's modified Eagle's medium
SF: synovial fluid
FBS: fetal bovine serum
DMSO: dimethyl sulfoxide
ALT: alanine aminotransferase
AST: aspartate aminotransferase
ALP: alkaline phosphatase
GGT, gamma-glutamyl transferase: LDH: lactate dehydrogenase
qPCR: quantitative polymerase chain reaction
GAPDH: glyceraldehyde 3-phosphate dehydrogenase.
